# Anatomical variants of dorsal scapular nerve in relation to the middle scalene muscle in Japanese population

**DOI:** 10.1097/MD.0000000000013349

**Published:** 2018-11-21

**Authors:** Shuhei Tetsu, Hayato Terayama, Ning Qu, Hajime Yamazaki, Reinii Sakamoto, Osamu Tanaka, Kaori Suyama, Motoyasu Takenaka, Toshiyasu Suzuki, Kou Sakabe

**Affiliations:** a Department of Anesthesiology; b Department of Anatomy; c Department of Palliative medicine, Tokai University School of Medicine 143, Shimokasuya, Isehara-shi, Kanagawa, Japan.

**Keywords:** adult Japanese cadavers, anatomical variation, dorsal scapular nerve, middle scalene muscle

## Abstract

Dorsal scapular nerve (DSN) block is often performed in Japanese pain clinics to treat neck pain and katakori (a unique symptom in Japanese population characterized by myofascial pain syndromes such as shoulder girdle pain). However, to the best of our knowledge, there are only a few studies regarding anatomical variations in DSN paths around the middle scalene muscle (MSM) in Japanese population. Thus, we conducted a cadaveric study to examine anatomical variations in DSN paths around the MSM in Japanese population.

DSN anatomies of 70 adult Japanese cadavers used for research and gross anatomy practice at the Tokai University School of Medicine between 2015 and 2016 were examined.

In all cadavers, DSNs originated from the brachial plexus (BP) and innervated the rhomboid major, rhomboid minor, and levator scapulae muscles via the MSM. Two types of DSN paths were observed: piercing-type (piercing the MSM) and anterior-type (running in front of the MSM). We surveyed all 140 sides in 70 Japanese cadavers; of these, 95 sides had piercing-type and 45 had anterior-type paths. Of the 70 cadavers, 42 had piercing-type and 17 had anterior-type paths on both the sides. In 9 cadavers, the left and right sides had piercing-type and anterior-type paths, respectively. In the other 2 cadavers, the right and left sides had piercing-type and anterior-type paths, respectively.

We found 2 distinct anatomical variants for DSN paths around the MSM in this Japanese cohort. Our results suggest that the rate of anterior-type DSN path is higher in Japanese population. Therefore, it is necessary to maintain caution while injecting anesthetic agents during a DSN block and the type of DSN should be considered.

## 1 Introduction

The dorsal scapular nerve (DSN) belongs to the anterior branch of the spinal nerve. The spinal nerves usually control ventral muscles.^[1]^ During development, muscles originate from dermomyotome of somite on the side of the neural tube and then move to their ultimate position.^[2]^ DSN undergoes developmental differentiation into 3 types piercing the middle scalene muscle (MSM) and then subsequently innervating the rhomboid and levator scapulae muscles. Many diseases such as thoracic outlet syndrome and myofascial pain syndrome (stiff neck) are associated with symptoms of discomfort, numbness, and dull pain from the neck to the shoulder. Neck pain has been reported to be caused by MSM strangling the DSN.^[3]^

According to the Japanese Pain Clinic textbook,^[4]^ DSN block is performed in Japanese pain clinics to treat neck pain and katakori (a unique symptom in Japanese population characterized by myofascial pain syndrome such as shoulder girdle pain). Reportedly, the incidence of katakori is higher in patients in Japan than in Europe or the United States. According to a Japanese government survey in 2017, the incidence of subjective katakori symptoms was 57.0 in men and 117.5 in women per 1000 individuals.^[5]^

Several types of DSN blocks exist, including the MSM, levator scapulae muscle, and rhomboid blocks.^[6]^ Additionally, it is possible to perform just levator scapulae and rhomboid muscle blocks and avoid the MSM block to prevent DSN injury. However, in cases with severe MSM symptoms, performing an MSM block is necessary. Therefore, the knowledge of anatomical variations in DSN paths around the MSM is crucial in the clinical settings to prevent complications associated with DSN block.

Generally, DSN originates from the fifth or fourth cervical spinal nerve and, after piercing the MSM, innervates the rhomboid major and minor muscles and the levator scapulae. Nguyen et al^[7]^ reported 3 types of DSN paths to the MSM: 1 piercing the MSM, 1 traveling anterior to the MSM, and 1 traveling posterior to the MSM. When creating an MSM block, the needle may injure DSN if the patient's DSN travels anterior to the MSM because the nerve is then exposed on the surface of the muscle. The posterior approach of a brachial plexus (BP) block (interscalene block) also carries the risk of DSN injury.^[8]^ To avoid unnecessary BP block complications, knowledge regarding the dissection of the MSM and regarding possible DSN variants is imperative. To the best of our knowledge,^[7,9,10]^ anatomical variations in DSN paths around the MSM have not been adequately reported in Japan. Thus, we conducted a cadaveric study to investigate anatomical variations in DSN paths around the MSM in Japanese population.

## 2 Methods

The study was conducted between 2015 and 2016 by examining 70 adult, Japanese, formalin-fixed cadavers used for research and gross anatomy practice at the Tokai University School of Medicine. This study was approved by the ethics committee of the Tokai University School of Medicine (No. 15R-105).

To study DSN path around the MSM, the skin on the back of the neck was carefully peeled and fat was dissected, ensuring that the blood vessels and nerves were not damaged. The clavicle was then removed to facilitate accurate observation. Subsequently, we dissected the neck (C1–7) and identified the anterior scalene muscle and MSM. The nerve emerging from the BP was identified as DSN. Next, the neck was placed in a prone position, and the nerve distribution was traced to the rhomboid major, rhomboid minor, and levator scapular muscles. We confirmed the nerve path to identify DSN. We examined DSN path around the MSM in detail and classified DSN paths into 3 types: piercing- (penetrating the MSM), anterior- (running in front of the MSM), and posterior-types (running behind the MSM). Table 1 shows^[7,9,10]^ previous case reports on DSN variants. Tubbs et al^[9]^ surveyed the dissection of DSN in American cadavers from a surgical point of view. Nguyen et al^[7]^ surveyed the anatomy of DSN and studied its oblique course with regards to the MSM. Izumi et al^[10]^ surveyed the dissection of DSN in Japanese cadavers to investigate the cause of thoracic outlet syndrome. Table 2 shows DSN types that were identified from cadaveric dissections in the present study. Statistical differences in the frequency of the 3 types of DSN paths between male and female cadavers (70 cadavers, 140 sides) were assessed using the *χ*^*2*^ (Yate's correction) and Fisher exact tests. All statistical analyses were conducted using the statistics test with a *P*-value of < .05 being statistically significant.

**Table 1:**
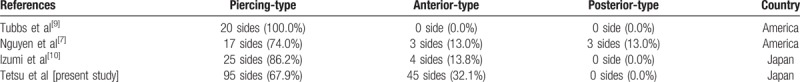
Comparison of anatomical variations in DSN paths identified in our study and in the literature.

**Table 2:**
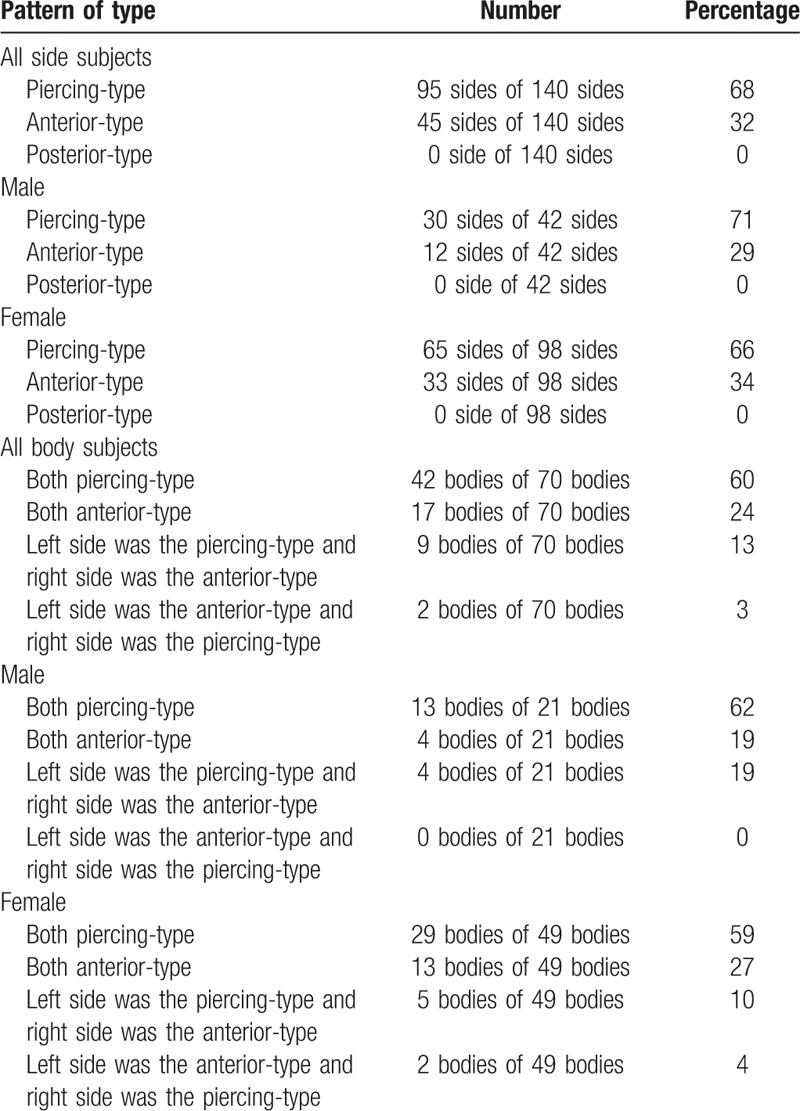
Anatomical variations in the type of DSN paths around the MSM in 70 Japanese cadavers (140 sides).

## 3 Results

**Figure 1:**
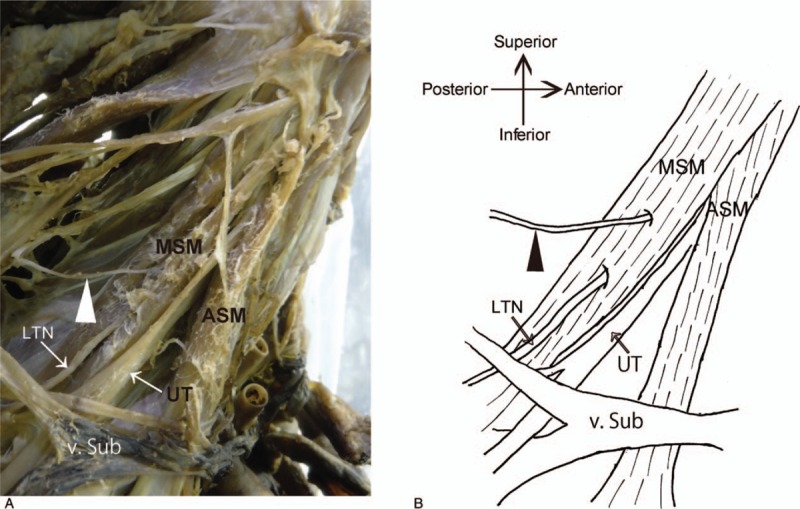
Photograph and schematic illustration of the piercing-type DSN path around the MSM. A, Anterolateral view of a formaldehyde-fixed, right neck of a 95-year-old man (cause of death: senility; No. 1938). B, Graphic schematic of the same view. A piercing-type DSN path piercing the MSM is identified. Black and white arrowheads point to DSN. ASM = anterior scalene muscle, DSN = dorsal scapular nerve, LTN = long thoracic nerve, MSM = middle scalene muscle, UT = upper trunk, v. sub = subclavian vein.

**Figure 2:**
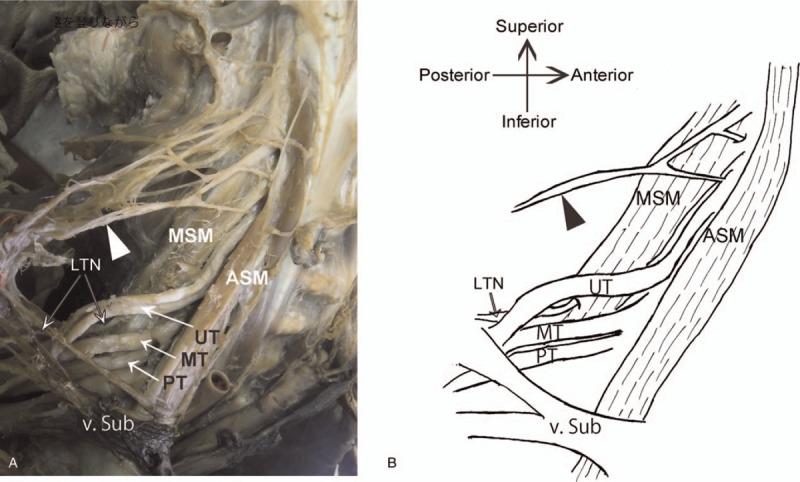
Photograph and schematic illustration of the anterior-type DSN path around the MSM. A, Anterolateral view of a formaldehyde-fixed, right neck of an 86-year-old woman (cause of death: gastrointestinal bleeding; No. 1838). B, Schematic representation of the same view. The anterior-type DSN path can be seen running in front of the MSM. Black and white arrowheads point to DSN. ASM = anterior scalene muscle, DSN = dorsal scapular nerve, LTN = long thoracic nerve, MSM = middle scalene muscle, MT = middle trunk, PT = posterior trunk, UT = upper trunk, v. sub = subclavian vein.

We observed 2 types of DSN paths: piercing-type (piercing the MSM) and anterior-type (running in front of the MSM). The piercing-type path was the most common path, originated from the fifth or fourth cervical spinal nerve, and pierced the MSM from the ventral to the dorsal side (Fig. 1). The anterior-type path was an anomaly originating in a similar manner as that of piercing-type of path, but it passed directly in front of the MSM (Fig. 2). In 70 Japanese cadavers, of the 140 sides, 95 had piercing- and 45 had anterior-type paths (Table 1). Moreover, of the 70 cadavers, 42 and 17 had piercing- and anterior-type paths on both the sides, respectively (Table 2). In 9 cadavers, the left and right sides had piercing- and anterior-type paths, respectively. In 2 cadavers, the right and left sides showed piercing- and anterior-type paths, respectively (Table 2). We found no significant differences in anatomical variations in DSN paths considering the whole body (*P* > .1) or the sides (*P* = .6933) between male and female cadavers (Table 2). Thus, our comparison of piercing- and anterior-type paths revealed no significant differences between men and women (neither in terms of side nor in terms of whole body variations). In the cadavers examined in this study, no posterior-type DSN paths (running behind the MSM) were observed.

The general consensus states that DSN pierces the MSM. However, in our study on anatomical variations of DSN, many cases with DSN running in front of the MSM without piercing were observed. Of the 140 sides in 70 cadavers, DSN pierced the MSM in 95 sides (67.9%), a rate lower than that previously reported (Table 1). In addition, as shown in Table 1, 86% of surveyed American cadavers had piercing-type DSN paths, whereas only 71% of our Japanese cadavers displayed this path type. Thus, the anterior-type path is more common in Japanese cadavers than in American cadavers, suggesting possible ethnic differences in the prevalence of DSN paths. Tubbs et al^[9]^ and Nguyen et al^[7]^ reported that in American cadavers, DSN pierced the MSM in all 20 sides (100.0%) and in 17 of 23 sides (74.0%) studied. Izumi et al^[10]^ investigated DSN path in Japanese cadavers to explore the cause of thoracic outlet syndrome and found that DSN pierced the MSM in 25 of 29 sides (86.2%).

## 4 Discussion

Our comparison of anatomical variations in DSN paths suggests that Japanese cadavers have a higher percentage of anterior-type paths than American cadavers. Additionally, the percentage of piercing-type paths was lower in our cadavers than that in American cadavers (Table 1). The results of Izumi et al^[10]^ slightly differed from those of our study possibly due to the different number of cadavers studied.

DSN blocks around the MSM are often performed in Japanese pain clinics to treat neck pain and katakori. To perform the block, a patient is placed in the supine position, and his/her face is positioned slightly toward the opposite of diseased side to improve the visibility of sternocleidomastoid muscles; the dorsal side edge of the sternocleidomastoid muscle is set as the insertion point. Once tenderness is confirmed, 1 to 3 mL of local anesthetic is locally injected. As the anterior-type path involves DSN piercing the MSM, DSN injury may occur during blocking. According to our results, the incidence of anterior-type paths is high in Japanese cadavers. Therefore, in the case of Japanese patients, practitioners need to consider the anatomical variation of DSN path when injecting an anesthetic during the DSN block.

Ultrasound-guided nerve blocks have become popular. Ultrasound allows for the visualization of 90% of DSN.^[11]^ However, ultrasound imaging of cervical nerves is more difficult than that limb nerves.^[12]^ As stated above, anatomical variations in DSN path need to be recognized for ensuring safer blocks. Moreover, the inability to visualize DSN using ultrasound imaging should prompt the consideration of an alternative block such as levator scapulae or rhomboid muscle block instead of an MSM block. The posterior approach of BP block also carries the risk of interscalene nerve (including the DSN and long thoracic nerves [LTNs]) injury.^[8]^ Although firm conclusions cannot be drawn regarding the LTN because it was not examined in the present study, the recognition of anatomical variations in DSN paths increases the probability of identifying DSN using ultrasound imaging and prevents nerve injury. Therefore, both piercing- and anterior-type paths as variations of DSN should be consideration while performing these types of blocks. As we used formalin-fixed cadavers, our ultrasound findings cannot be extrapolated to actual patients; in the future, we will conduct studies with fresh cadavers and compare ultrasound findings. This will improve the possibility of visual recognition, and the difference in the appearance of piercing- and anterior-type paths may become clear.

In this study, we reported 2 types of DSN paths in Japanese population differing with respect to the DSN path around the MSM. In addition, comparison with other reports on anatomical variations in DSN paths indicate a possible association with ethnic groups, and this is may help explain the causality of katakori.

Although our results cannot be used to propose new clinical guidelines or applications to the head and neck operations or while performing BP blocks (interscalene or supraclavicular), the recognition of these anatomical variants warrants further studies that can prevent complications.

In conclusion, we observed piercing- and anterior-type DSN paths in the studied Japanese cadavers. Our results showed that the incidence of anterior-type DSN paths is high in the Japanese population. This can be useful when considering DSN blocks, even for ultrasound-guided procedures.

## Acknowledgments

The authors would like to thank Mr. Noriyuki Kosemura, Ms. Kyoko Endo, and Ms. Yuko Furuya from the Tokai University School of Medicine, Kanagawa, Japan for their excellent secretarial support. We would like to thank Enago and Editage for English language editing.

## Author contributions

ST, HT, and KS designed the study and wrote the initial draft of the manuscript. ST dissected all cadavers. ST, HT, NQ, and HY observed and confirmed all findings. ST and HT contributed to the analysis and interpretation of data and assisted in the preparation of the article. All other authors contributed to data collection and interpretation and in critically reviewing the article. All authors approved the final version of the article and are accountable for all aspects of the study; they will also ensure that questions related to the accuracy or integrity of any part of the work are appropriately investigated and resolved.

**Conceptualization:** Shuhei Tetsu, Hayato Terayama, Ning Qu, Hajime Yamazaki.

**Data curation:** Shuhei Tetsu, Hayato Terayama, Ning Qu, Hajime Yamazaki, Reinii Sakamoto.

**Formal analysis:** Shuhei Tetsu, Hayato Terayama, Ning Qu, Hajime Yamazaki.

**Investigation:** Shuhei Tetsu, Hayato Terayama, Ning Qu, Hajime Yamazaki, Reinii Sakamoto.

**Methodology:** Shuhei Tetsu, Hayato Terayama, Ning Qu, Hajime Yamazaki.

**Project administration:** Shuhei Tetsu, Hayato Terayama, Hajime Yamazaki.

**Resources:** Shuhei Tetsu, Hayato Terayama, Ning Qu.

**Supervision:** Hayato Terayama, Ning Qu, Hajime Yamazaki, Reinii Sakamoto, Osamu Tanaka, Kaori Suyama, Motoyasu Takenaka, Toshiyasu Suzuki, Kou Sakabe.

**Validation:** Shuhei Tetsu, Hayato Terayama, Ning Qu.

**Visualization:** Shuhei Tetsu, Hayato Terayama, Ning Qu.

**Writing – original draft:** Shuhei Tetsu, Hayato Terayama.

**Writing – review & editing:** Shuhei Tetsu, Hayato Terayama, Ning Qu, Kou Sakabe.
